# Extraction of Antioxidants from Winemaking Byproducts: Effect of the Solvent on Phenolic Composition, Antioxidant and Anti-Cholinesterase Activities, and Electrochemical Behaviour

**DOI:** 10.3390/antiox9080675

**Published:** 2020-07-28

**Authors:** María José Jara-Palacios, Sandra Gonçalves, Francisco J. Heredia, Dolores Hernanz, Anabela Romano

**Affiliations:** 1 Department of Analytical Chemistry, Facultad de Farmacia, Universidad de Sevilla, 41012 Sevilla, Spain; mjara@us.es; 2 MED—Mediterranean Institute for Agriculture, Environment and Development, Universidade do Algarve, Faculdade de Ciências e Tecnologia, Campus de Gambelas, Ed. 8, 8005-139 Faro, Portugal; smgoncalves@ualg.pt (S.G.); aromano@ualg.pt (A.R.); 3 Food Colour & Quality Laboratory, Área de Nutrición y Bromatología, Facultad de Farmacia, Universidad de Sevilla, 41012 Sevilla, Spain; heredia@us.es

**Keywords:** grape pomace, UHPLC, DPPH, ORAC, acetylcholinesterase, butyrylcholinesterase, electrochemistry

## Abstract

Extraction solvent is a very important factor in the recovery of antioxidants from natural matrices. In this study, the effect of three solvents (ethanol, ethanol/water and water) on the phenolic composition, antioxidant and anti-cholinesterase activities and electrochemical behaviour of four winemaking byproducts (seeds, skins, stems, and pomace) was evaluated. Phenolic composition was determined by the Folin–Ciocalteu method and ultra-high-performance liquid chromatography (UHPLC), antioxidant activity by the capacity to scavenge 2,2-diphenyl-1-picrylhydrazyl and hydroxyl radicals, anti-cholinesterase activity by the Ellman’s method, and electrochemical behaviour by cyclic voltammetry. Eight phenolic compounds were quantified with higher content in water/ethanol extracts (e.g., epicatechin in pomace: 17 mg/100 g vs. 7 and 6 mg/100 g in ethanol and water extracts, respectively), although there were some exceptions (e.g., gallic acid in seeds was most abundant in water extracts). Moreover, the highest total phenolic content (TPC) and antioxidant activity were found in ethanol/water extracts (between 2 and 30-fold the values of the other extracts). Overall, the most active extracts in inhibiting both acetylcholinesterase (AChE) and butyrylcholinesterase (BChE) enzymes were ethanol/water and ethanol extracts from seeds (between 31.11 and 53.90%). The electrochemical behaviour allowed for differentiating the extracts depending on the solvent and the byproduct. Our findings indicate that winemaking byproducts represent a source of phenolic compounds with antioxidant and anti-cholinesterase activities and suggest that cyclic voltammetry is a promising technique to evaluate the phenolic extraction process from these byproducts.

## 1. Introduction

Antioxidants are artificial or natural chemical substances that delay the oxidation of proteins, DNA and lipids, due to their properties, and can counteract oxidative stress [1]. Oxidative stress causes biomolecular damage due to the attack of reactive species, mainly reactive oxygen species (ROS), on the cells and tissues of the living organisms [2,3]. This oxidative damage is related to the physiopathology of many diseases, such as cancer, atherosclerosis, cardiovascular, metabolic and neurodegenerative diseases, and brain ageing [2,3,4,5,6].

Neurodegenerative diseases are particularly related to oxidative stress [7,8]. These diseases are also characterised by alterations in the concentration and activity of an important neurotransmitter namely acetylcholine [9]. A specific treatment is based on increasing the concentration of acetylcholine by inhibiting the activity of acetylcholinesterase (AChE) and butylcholinesterase (BChE), which are key enzymes in the breakdown of this neurotransmitter [10].

Phenolic compounds from plants have shown simultaneously antioxidant and anticholinesterase properties and have been suggested to protect neurons from oxidative stress [11,12,13,14]. Therefore, they are of great interest for the prevention and treatment of these neurological disorders [15]. Winemaking byproducts are important natural sources of phenolic compounds [16,17,18] with several applications. There is a growing demand for these byproducts due to increased use of natural compounds and the necessity of an efficient management of these byproducts for a sustainable wine industry [19]. They can also be incorporated in dietary supplements or be used to develop functional foods [20].

Extraction is a very important step in the recovery of phenolic compounds from winemaking byproducts [18]. It is important to use solvents innocuous to human health when the obtained phenolic extracts are used in the elaboration of functional foods, cosmetics, and pharmaceutical and dietary supplements. In addition, extraction solvents must be environmentally friendly. Water and ethanol are the most used conventional solvents [21] and are considered GRAS (Generally Recognised as Safe) [22].

Several studies with extracts from winemaking byproducts rich in phenolic compounds have been carried out for evaluation of antioxidant properties [23,24,25]. Although in vitro antioxidant activity of winemaking byproducts has been widely studied by different spectrophotometric methods, studies on the in vitro anti-cholinesterase properties of these products are still scarce. The recent studies on the cholinesterase inhibitory activities of red wine and seeds, skins and flesh from *Vitis vinifera* fruits can be highlighted [9,26]. These studies indicated that wine, seeds and skins had high anticholinergic activity. The spectrophotometric methods are currently being questioned because a single in vitro method cannot reflect the total antioxidant potential, and, due to discrepancy in the in vitro and in vivo antioxidant results [27,28]. The cyclic voltammetry technique has been proposed as a good alternative to estimate the total antioxidant potential of winemaking byproducts and the electrochemical behavior of antioxidant compounds has been correlated with the results of in vitro and in vivo biological methods [17,29,30].

The aim of this work was to extract phenolic compounds from different winemaking byproducts using innocuous and environmentally friendly solvents. The phenolic composition, the in vitro antioxidant and anti-cholinesterase activities of those extracts was evaluated. In addition, their electrochemical behavior was studied and a correlation analysis between results was performed.

## 2. Materials and Methods

### 2.1. Standards and Reagents

Sodium carbonate (Na_2_CO_3_) and Folin–Ciocalteau reagent were acquired from VWR (Leuven, Belgium). Methanol, ethanol, 2,2-diphenyl-1-picrylhydrazyl (DPPH), galanthamine, AChE (Electric-eel, EC 3.1.1.7, Type VIS), acetylthiocholine iodide (ATCI), BChE (EC 3.1.1.8), butyrylthiocholine iodide (BTCI) and 5,5’-dithiobis-[2-nitrobenzoic acid] (Ellman’s reagent or DTNB) were purchased from Sigma-Aldrich (Steinheim, Germany). Fluorescein (FL), 6-hydroxy-2,5,7,8-tetramethylchromane-2-carboxylic acid (Trolox) and 2,2-azobis(2-methylpropionamidine) dihydrochloride (AAPH) were acquired from Acros Organics (Geel, Germany).

Formic acid, high-performance liquid chromatography (HPLC)-grade acetonitrile, acetic acid and sodium acetate were obtained from Panreac (Barcelona, Spain). Gallic acid, protocatechuic acid, catechin, epicatechin and quercetin 3-*O*-rutinoside (rutin) were purchased from Sigma-Aldrich (Madrid, Spain) and quercetin 3-*O*-glucoside and kaempferol 3-*O*-glucoside from Extrasynthese (Genay, France).

### 2.2. Samples

Four types of samples from white winemaking byproducts (*Vitis vinifera* var. Zalema) were analysed: grape pomace (including a mixture of seeds, skins, stems and rests of pulp), and skins, seeds, and stems separated from this pomace. The samples were washed with distilled water, freeze-dried and ground (dry byproduct) for subsequent extraction.

The byproducts were supplied by “Cooperativa Nuestra Señora del Socorro” winery, belonging to Condado de Huelva Designation of Origin (Huelva, southwestern Spain).

### 2.3. Phenolic Extraction

The phenolic extraction was carried out as previously described [16] with some modification and using three different solvents: (1) ethanol, (2) ethanol/water in a 1:1 mixture and (3) water. The dry sample (5× *g*) was homogenised in 25 mL of the solvent with agitation for 1 h and then centrifuged (4190× *g* for 15 min). The supernatant was collected, and the residue submitted to the same process twice. The liquid supernatants were combined (phenolic extract) and stored until analysis. The extractions were carried out in quadruplicate. Three replicates from each byproduct were concentrated to dryness in a rotary evaporator and redissolved in the amount of solvent necessary to reach a concentration of 50 mg/mL. A fourth replicate was only concentrated to determine the extraction yields expressed in percentage (%).

### 2.4. Total Phenolic Content

The total phenolic content (TPC) was determined using the Folin–Ciocalteu assay with some modifications [31]. Firstly, 200 μL of 10% (*v/v*) Folin–Ciocalteu reagent, 100 μL of each phenolic extract and 800 μL of 700 mM Na_2_CO_3_ were mixed and incubated in the dark for 2 h at room temperature. Secondly, the absorbance of 200 μL of reaction mixture was measured at 765 nm in a microplate reader. Gallic acid was employed as a calibration standard and results were expressed as mg of gallic acid per 100 *g* of dry byproduct (mg GAE/100 g).

### 2.5. Chromatographic Analyses

The chromatographic analyses were carried out in an Agilent 1290 chromatograph (Agilent Technologies, Palo Alto, CA, USA) equipped with a diode-array detector, which was set to scan from 200 to 770 nm, and a C18 Eclipse Plus 120 column (1.8 µm, 50 × 2.1 mm). The analysis conditions were established following the previously described method [32]. The solvents were 0.01% formic acid in water (solvent A), and acetonitrile (solvent B) at the following gradient: 0–5 min, 5% B linear; 5–20 min, 50% B linear; 20–25 min, 100% A linear, washing and re-equilibration of the column. The flowrate was 1.0 mL/min, and the temperature of the column was set at 25 °C. The phenolic extracts (2 mL) were concentrated to dryness, re-dissolved in 1 mL of solvent A, filtered (0.45 μm filter) and injected (1 µL) into the chromatograph.

Phenolic compounds were identified by their retention time and UV-vis spectra, as well as by comparison with our data library and standards when available. The quantification was carried out by external calibration from the areas of the chromatographic peaks, as previously described [32].

Three replicates from each sample were analysed, and the results were expressed as mg of phenolic compound per 100 *g* of dry byproduct (mg/100 g).

### 2.6. DPPH Free Radical Scavenging Assay

The capacity of samples to scavenge DPPH radicals was evaluated according to the previous protocol with some modifications [31]. Samples were diluted to an adequate concentration in relation to the Trolox standard (a dilution that produced between 20–80% inhibition of the blank absorbance, which was different depending on the type of extract). Methanol was used as a negative control (blank) and Trolox as a positive control (standard). Firstly, 300 μL of 90 μM DPPH methanolic solution was added to 30 μL of diluted sample, Trolox (0.025–0.3 mM) or methanol, and the mixtures were diluted with 570 μL of methanol in eppendorf tubes. The reaction mixtures were incubated in the dark at room temperature and, after 30 min, the reduction of DPPH radicals was determined by measuring absorbance at 515 nm in a spectrophotometer. Three replicates from each sample were analysed and the radical scavenger activity was expressed as mmol of Trolox equivalents per 100 *g* of dry byproduct (mmol TE/100 g).

### 2.7. Oxygen Radical Absorbance Capacity (ORAC) Assay

The ORAC was evaluated as previously described with some modifications [31]. Samples were diluted to an adequate concentration in relation to the Trolox standard (dilution was different for each type of extract). FL was used as the fluorescent probe and AAPH as the peroxyl radical generator. Trolox was used as a positive control (standard) and phosphate buffer (75 mM, pH 7) as a negative control (blank). In each well of a black 96-well microplate, 150 μL of FL (80 mM) and 25 μL of diluted sample, Trolox (6–50 μM) or phosphate buffer were placed, and the reaction was initiated by adding 25 μL of AAPH (140 mM) to each well after incubating for 10 min at 37 °C. The reduction in fluorescence was determined by reading fluorescein excitation at 485 nm and emission at 530 nm every minute for 90 min in a microplate reader. Three replicates from each sample were analysed. The ORAC values were calculated using the area under the curve (AUC) and the results were expressed as mmol of Trolox equivalents per 100 *g* of dry byproduct (mmol TE/100 g).

### 2.8. In Vitro Cholinesterases Inhibition Assay

The AChE and BChE inhibitory activities were evaluated as described by Ellman’s method [33]. Firstly, 3 mM DTNB, 15 mM substrate (ATCI or BTCI), 100 mM phosphate buffer (pH 8.0) and phenolic extract (2.5 mg/mL), buffer or galanthamine (standard inhibitor) were mixed in a 96-well microplate. Then, AChE or BChE (0.28 U/mL) were added and the absorbance read at 405 nm immediately and after 5 min. Finally, the enzyme activity was calculated as a percentage of the velocities compared to that of the assay using buffer without any inhibitor. Three replicates from each sample were analysed. The results were expressed as the percentage inhibition (%).

### 2.9. Electrochemical Assays

The electrochemical behaviour of samples was evaluated by cyclic voltammetry as previously described [34]. A potentiostat/galvanostat (AUTOLAB model PGSTAT 302 N, Metrohm Autolab B.V., Utrecht, The Netherlands) controlled by a General Purpose Electrochemical System (GPES) software (Metrohm Autolab B.V., Utrecht, The Netherlands) was used.

The phenolic extracts were appropriately diluted in 0.1 M sodium acetate-acetic acid buffer at pH 3.6 and transferred into a glass electrochemical cell. The measurements were carried out using a glassy carbon working electrode, a platinum auxiliary electrode and an Ag/AgCl reference electrode. The cyclic voltammogram scans were made from 0.0 to 1.0 V at a scan rate of 100 mV/s.

The electrochemical parameters extracted from the cyclic voltammetry curves were the anodic current area (Q), the anodic peak potential (E_p,a_), the anodic peak current (I_p,a_), the cathodic peak potential (E_p,c_) and the cathodic peak current (I_p,c_) of the two main peaks in the cyclic voltammograms. Q represents the integrated area of the cyclic voltammogram for scans taken from 0.0 to 1.0 V. All the samples were analysed in triplicate.

### 2.10. Statistical Analysis

The Statisticav.8.0 software was used to perform all statistical analyses. The data were examined to establish if total phenolic content, individual phenolic compounds, antioxidant activity (DPPH and ORAC values), anti-cholinesterase properties (AChE and BChE inhibition percentages) and electrochemical parameters (Q, E_p,a_, I_p,a_, E_p,c_ and I_p,c_) differed significantly between ethanol, ethanol/water and water extracts from each type of winemaking byproduct. The data were presented as the mean ± standard deviation (SD) and were processed by one-way analysis of variance (ANOVA). Significant differences between means were identified using Tukey’s New Multiple Range Test (*p* < 0.05). General discriminant analyses (GDA) were performed to classify the extracts according to the electrochemical behaviour.

## 3. Results

### 3.1. Extraction Yields

**Figure 1:**
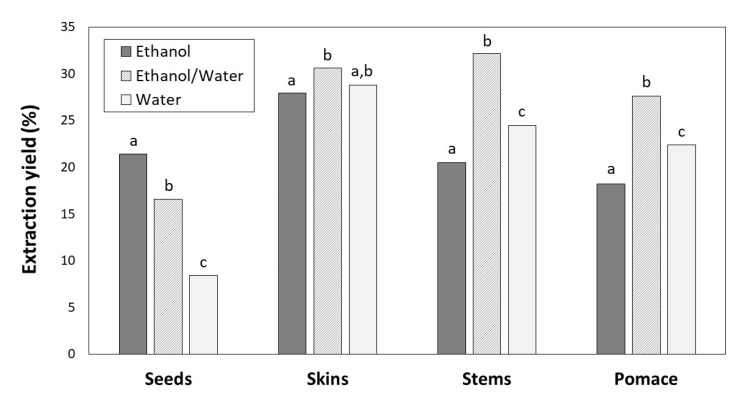
Extraction yields for seeds, skins, stems and pomace depending on the solvent (ethanol, ethanol/water and water). For the same byproduct, different letters indicate significant differences (*p* < 0.05) between ethanol, ethanol/water and water extracts by Tukey’s test.

The extraction yields expressed as weight of phenolic extract relative to the weight of the initial sample (dry byproduct) ranged from 8.41% to 32.46% and depended on the solvent and type of byproduct (Figure 1).

For skins, stems and pomace, the highest extraction yields were achieved with ethanol/water (30.58, 32.16 and 27.64%, respectively) followed by water (28.81, 24.49 and 22.42%, respectively) and ethanol (27.91, 20.47 and 18.23%, respectively). However, the greatest yield for seeds was found with ethanol (21.40%), followed by ethanol/water (16.57%) and water (8.41%).

### 3.2. Total Phenolic Content

**Figure 2:**
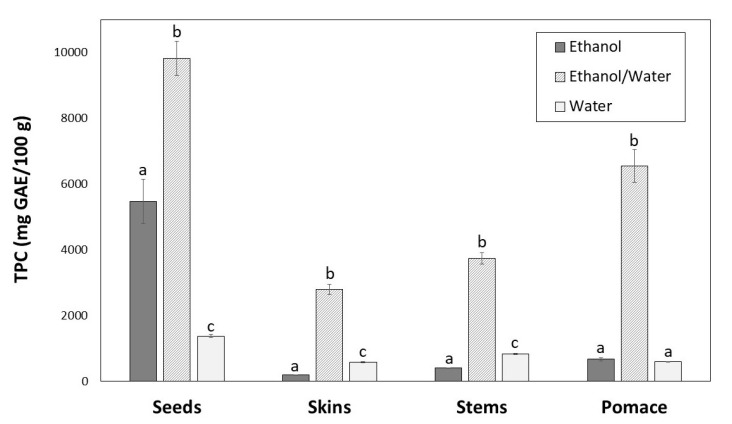
Total phenolic content of seeds, skins, stems and pomace extracts obtained with different solvents. For the same byproduct, different letters indicate significant differences (*p* < 0.05) between ethanol, ethanol/water and water extracts by Tukey’s test.

The TPC of winemaking byproducts extracts is shown in Figure 2. Significant differences (*p* < 0.05) were found depending on the extraction solvent. For all byproducts, the highest content was achieved with ethanol/water (9839.86, 6554.16, 3745.77 and 2797.67 mg GAE/100 g, for seeds, pomace, stems and skins, respectively).

The greatest TPC was found for seeds with ethanol/water (Figure 2), which showed lowest extraction yield (Figure 1). Regarding ethanol and water solvents, the TPC were different for each byproduct. For skins and stems water provided the better results, and for seeds and pomace ethanol was more efficient, although the differences were not significant in the case of pomace extracts (Figure 2).

### 3.3. Determination of Phenolic Compounds

Eight individual phenolic compounds belonging to three phenolic groups (phenolic acids: gallic and protocatechuic acids; flavanols: catechin, epicatechin and proanthocyanidin B1; and flavonols: quercetin 3-*O*-rutinoside, quercetin 3-*O*-glucoside and kaempferol 3-*O*-glucoside) were determined in order to evaluate the effects of the extraction solvent on the qualitative and quantitative phenolic profile of each winemaking byproduct extract. The phenolic compounds identified and quantified in each winemaking byproduct are presented in Table 1.

Regarding flavanols and flavonols, the best results for all byproducts were observed using ethanol/water as extractant. The flavanols, the most abundant compounds in seeds, were largely extracted with ethanol/water (33.04, 40.04 and 29.36 mg/100 g of catechin, epicatechin and proanthocyanidin B1, respectively); although ethanol and water also provided high contents (with values between 18.09 and 27.47 mg/100 g). This tendency was also observed for the pomace. The flavanols extracted with water from skins and with ethanol from stems could not be quantified because they were lower than the limits of quantification. 

The highest flavonol content was found for skins extracted with ethanol/water being the main compound quercetin 3-*O*-glucoside (48.85 mg/100 g), about 1.5-fold and 92-fold greater than in ethanol and water extracts, respectively. For all byproducts, the amount of flavonols extracted with ethanol and water was relatively low, except for skins extracted with ethanol that, as mentioned, provided contents between 5.11 and 28.97 mg/100 g (Table 1). In general, water was not a suitable solvent for flavonols from winemaking byproducts.

Ethanol/water was also the best solvent to extract phenolic acids, although some exceptions were observed. For seeds, great amounts of gallic acid were obtained using water (41.45 mg/100 g), nearly 2.5-fold higher than for ethanol/water (16.66 mg/100 g) and about 5.5-fold higher than for ethanol (7.42 mg/100 g). For stems, water was also the best solvent to extract gallic acid (14.98 mg/100 g), nearly 1.2-fold and 4.0-fold higher than for ethanol/water and ethanol, respectively; although, this was not significantly different (*p* > 0.05) when compared with ethanol/water (12.36 mg/100 g).

### 3.4. Antioxidant Activity

All the byproducts demonstrated a free-radical scavenging capacity and the extracts obtained with ethanol/water showed the highest antioxidant activity for both assays (Table 2). 

For DPPH assay, the antioxidant activity is in agreement with the results of TPC for each sample. In contrast, the antioxidant activity measured by ORAC assay only agreed with TPC for skins and stems. All seeds extracts were significantly better (*p* < 0.05) at neutralizing DPPH radicals than other byproducts, showing that skin extracts hold the lowest values. However, all extracts from skins displayed a greater scavenging capacity of peroxyl radicals (measured by ORAC assay) than extracts from seeds (Table 2). The lowest ORAC values were found with ethanol extracts in all byproducts.

### 3.5. In Vitro Cholinesterases Inhibition

**Figure 3:**
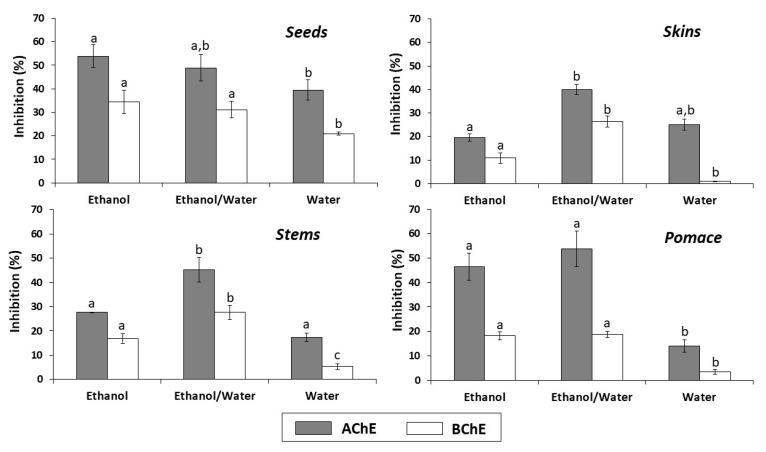
In vitro cholinesterase inhibitory activity of seeds, skins, stems and pomace extracts (2.5 mg/mL) obtained with different solvents. In each graph, for acetylcholinesterase (AChE) or butyrylcholinesterase (BChE), the letters indicate significant differences (*p* < 0.05) between ethanol, ethanol/water and water extracts by Tukey’s test.

The results of the anti-cholinesterase activity of the different extracts from all types of byproducts are shown in Figure 3. Overall, the ethanol/water and ethanol extracts of all byproducts, at the concentration evaluated (2.5 mg/mL), exhibited the highest AChE and BChE inhibition percentages. The ethanol extract from seeds was the most effective AChE and BChE inhibitor (53.90 and 34.51%, respectively). On the other hand, the lowest inhibition percentages were observed with the water extracts from skins, pomace and stems for BChE (0.90, 3.49 and 5.39%, respectively).

### 3.6. Electrochemical Behavior by Cyclic Voltammetry

The cyclic voltammograms had three anodic peaks (positive current) and one cathodic peak (negative current). In all samples, only one of the anodic peaks is well defined. Therefore, to simplify the interpretation of the electrochemical information, results will be discussed based on one single anodic peak (peak a), the cathodic peak (peak c) and the anodic current area (Q). The electrochemical parameters (Q, E_p,a_, I_p,a_, E_p,c_, I_p,c_) extracted from the voltammograms of the byproduct extracts are presented in Table 3. For all the byproducts, the peaks a and c had E_p,a_ and E_p,c_ values ranging between 0.41-0.49 and between 0.27-0.45 V, respectively.

**Figure 4:**
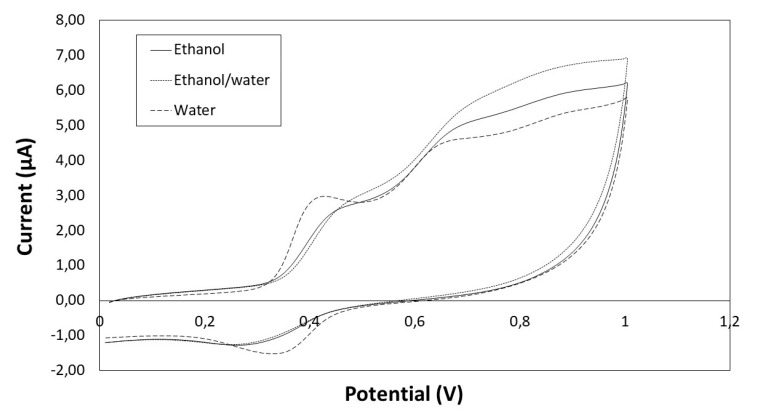
Cyclic voltammogram of seeds extracts obtained with different solvents (ethanol, ethanol/water and water).

The cyclic voltammograms of phenolic extracts from seeds for scan from 0 to 1 V are shown in Figure 4, and the peaks can be observed: peak a between 0.41–0.48 V and peak c between 0.30–0.35 V (depending on the solvent).

Regarding the parameter Q, although the higher value was found for ethanol/water extract, there were no significant differences depending on the solvent. However, the I_p,a_ value for water extracts (3.00 μA) was significantly higher (*p* < 0.05) than for ethanol/water (2.79 μA) and ethanol extracts (2.66 μA), which is probably due to the high content of gallic acid in the water extracts (Table 3). The E_p,a_ value for peak a was 0.41 V, which coincides with the E_p,a_ value for gallic acid (data not shown). On the other hand, the peak a for ethanol/water and ethanol extracts was mainly due to the high concentration of flavanols (E_p,a_ = 0.48 V and E_p,a_ = 0.46 V, respectively).

The cyclic voltammogram of skin ethanol/water extract showed higher Q and I_p,a_ values (*p* < 0.05) than water and ethanol extracts (Table 3), which is indicative of a greater amount of phenolics, mainly flavonols (E_p,a_ = 0.49 V). For stem and pomace, the Q values were significantly different (*p* < 0.05) depending on the extraction solvent. For both byproducts, ethanol/water extracts showed the highest Q values; however, the I_p,a_ value from pomace was greater for ethanol extracts. Peaks a of ethanol/water extracts from stems and pomace were related to flavanols and flavonols. Peak a of water extracts from stems was mainly influenced by gallic acid content (E_p,a_ = 0.42 V).

Peak b is related to the anodic peak on a reversible electrode reaction. This peak showed differences for I_p,c_ and E_p,c_ depending on the extraction solvent and the type of byproduct (Table 3). Regarding seeds and pomace, these electrochemical parameters indicated significant differences (*p* < 0.05) between water extracts and the other two extracts. Moreover, the skin extracts were differentiated by the significant differences between these parameters. In regard to E_p,c_ for stems, the values were not significantly different depending on the solvent, but I_p,c_ values indicated significant differences.

Finally, various GDA were carried out to establish whether the three types of extracts can be classified according to electrochemical behaviour. For this purpose, all variables were considered: the electrochemical parameters (Q, E_p,a_, I_p,a_, E_p,c_, I_p,c_), TPC, antioxidant activity (DPPH and ORAC), anti-cholinesterase activity (AChE and BChE), and eight phenolic compounds. First, all variables were included in the analysis and a 100% correct classification was obtained. Secondly, a GDA was carried out only for electrochemical variables and the percentage of correct classification was (86.00%). An increase in the classification percentage was observed when variables of anti-cholinesterase activity were included (97.22%). However, if only the TPC and antioxidant activity variables are considered, the correct classification is lower (58.33%).

## 4. Discussion

Winemaking is an activity that leads to the generation of large amounts of byproducts, such as grape pomace, rich in phenolic compounds. White grape pomace contains higher proportions of phenolic compounds than red grape pomace, because, in white winemaking grape must is not fermented with the solid parts and then phenolic compounds remain in them. As a consequence, there is increasing interest in the exploitation and valorization of those white winemaking byproducts.

The phenolic composition of extracts from white winemaking byproducts depends on the extraction method, and the factors to consider are sample pre-treatment, solvent, solvent-solid ratio, extraction mode, temperature, and time [18]. Solvent is one of the most relevant factors in extraction efficiency [19,35]. Although methanol/water is the most effective mixture for phenolic compounds extraction [16], it is important to use environmentally friendly, innocuous to human health and economic solvents. In this sense, GRAS solvents such as ethanol and water are very interesting. In this study, ethanol, ethanol/water and water were used as extraction solvents, and for all byproducts, the highest content was achieved with ethanol/water. In a previous report, these three solvents were used to recover polyphenols from vinification byproducts, and a mixture of ethanol/water also provided the best results [21]. In another study, the efficiency of water and aqueous ethanol at different concentrations (50%, 70% and 96%) was tested to extract phenolics from grape seeds, and, similar to those obtained in this work, the best results were achieved with 50% aqueous ethanol [36].

There is no single solvent able to extract all the phenolic compounds from a winemaking byproduct simultaneously. Seeds, skins, stems and pomace are different plant materials composed by different phenolic compounds with distinct polarities. Moreover, the solubility of phenolic compounds is influenced by various aspects [37]: (a) their chemical nature, compounds from a simple to polymerised structure; (b) the plant materials; and (c) the polarity of the solvent used. The extraction yields also influence phenolic extraction. In general, the extraction yields for seeds were lower than those for other byproducts, which could be due to their solid cellular structure [38]. The same reason can explain the extraction yield from pomace that was composed by 26.95% seeds, 47.55% skins and 25.50% stems. Solvents with medium polarity are generally most efficient for extracting phenolic compounds; therefore, the combination of water and organic solvents is more effective than water or pure ethanol. Water also extracts other compounds (e.g., organic acids, sugars, soluble proteins), which could interfere in phenolic quantification. On the other hand, highly pure organic solvent as ethanol at 100% could dehydrate the plant cells and not allow the diffusion of phenolic compounds, decreasing the extraction yield [36,39,40]. In this work, the greatest TPC was found for seeds with ethanol/water (Figure 2), which showed lowest extraction yield (Figure 1). This fact can be explained by the higher accumulation of phenolic compounds in seeds than in other byproducts [38].

Phenolic compounds that are present in the white winemaking byproducts mainly belong to three phenolic groups (flavanols, flavonols and phenolic acids) [16]. The most abundant individual compounds of each phenolic group were selected to study the effect of the extraction solvent (Table 1). The phenolic profile of each byproduct extract was strongly influenced by the extraction solvent. In general, water was not a suitable solvent to extracts flavonols and ethanol/water was the most efficient solvent to extract all phenolic compounds from all byproducts, except gallic acid from seeds and stems. Seeds and stems are very rich in gallic acid, which is a highly hydroxylated and hydrophilic compound easily soluble in water. Regarding flavonols, ethanol provided higher extraction yields than water in skins, seeds and pomace, possibly because highly methoxylated and lipophilic compounds, such as quercetin, show good diffusion in lower polarity solvents as pure ethanol [21,41].

The antioxidant activity of the different winemaking byproducts can be evaluating by chemical methods based on different mechanisms by which plant antioxidants can exert their action. In this work, DPPH, a hydrogen atom transfer-based method, and ORAC, a single electron transfer-based method, were applied. Some authors have reported that the mixture of water:ethanol (1:1) is a more suitable solvent than water and ethanol individually for the extraction of antioxidant compounds from grape pomace and seeds [21,42], which is in accordance with our results.

Skins show a greater scavenging capacity of peroxyl radicals (ORAC assay) than extracts from seeds (Table 2) [32]. This could be due to the fact that skins are much richer in flavonols than in other compounds and these compounds contribute greatly to the peroxyl radicals scavenging capacity. The “Zalema” variety showed the highest concentration in flavonols in comparison to other varieties, which strongly influenced the antioxidant activity [34]. In this study, the antioxidant activity of the phenolic extracts from the same winemaking byproducts extracted with methanol was determined by the ABTS radical scavenging assay. The antiradical activity was higher for seed extracts and lowest for skin extracts, which is in accordance with the results obtained in this study for DPPH assay.

In addition to antioxidant activity, the phenolic compounds present anti-cholinesterase activity. Extracts from natural products have shown that anti-cholinesterase activity has been associated with the phenolic compounds [13,14,43,44]. The few studies on the cholinesterases inhibition of wine and winemaking byproducts indicate that this activity is related to the type and structure of phenolic compounds [9,26]. In this study, the AChE than BChE inhibition observed for all the extracts from all types of winemaking byproducts is in accordance with previous results [9]. However, these indicated that skins had greater AChE and BChE inhibition than seeds, which does not agree with our results. These differences can be due to different grape type (white or red grapes) or variety. Anti-cholinesterase assays have not been previously performed by white winemaking byproducts and our results show a good activity compared to galanthamine. This is a natural product belonging to the Amaryllidaceae family of alkaloids used as positive control in the laboratory and has been reported as a compound that inhibits the activity of AChE and BChE [45]. Galanthamine inhibited 55.86% of the activity of AChE and 15.26% of BChE. A previous study reported that galanthamine was more active against AChE than BChE since it is a selective AChE inhibitor [13].

Further studies are required to elucidate the specific phenolic compounds, present in each type of winemaking byproduct, which are responsible for these anti-cholinesterase effects and the mechanisms involved; although, our data support the idea that major polyphenols present in the extracts and a combination of all phenolic compounds are responsible.

Electroanalytical methods are also used to measure the antioxidant activity. Therefore, the electrochemical behaviour of phenolic extracts from the four types of winemaking byproducts was evaluated by cyclic voltammetry. This technique was previously applied to measure the antioxidant potential to correlate electrochemical parameter values to lipid peroxidation inhibition, and the results indicated a good correlation. As previously suggested [17], the anodic and cathodic peak potentials (E_p,a_ and E_p,c_) can be related to the type of phenolic compounds and the anodic and cathodic peak currents (I_p,a_) to the concentration of the phenolic compounds. In this work, the significant differences found between the electrochemical parameter values indicate a different phenolic composition for different extracts from each type of winemaking byproduct. The statistical analysis was performed in order to establish whether the phenolic extraction can be evaluated by the electrochemical behavior. Although the electrochemical parameters evaluated by cyclic voltammetry do not allow for a correct classification of 100%, the percentage (86%) is much higher than using the traditional spectrophotometric methodology (Folin–Ciocalteu and DPPH, and ORAC assays) (58.33%). 

## 5. Conclusions

In this study, three types of extracts obtained from four types of winemaking byproducts were analysed. The ethanol/water extracts had higher phenolic content (TPC and individual phenolic compounds), free-radical scavenging capacity and anti-cholinesterase activity than ethanol and water extracts. In general, seeds showed better results than pomace, stems and skins. 

Differences in the electrochemical parameters were found depending on the extraction solvent and the type of byproduct. Therefore, these results suggest that cyclic voltammetry allows for evaluating the antioxidant potential and the effect of the extraction solvent on the phenolic composition of the different winemaking byproducts.

This work presents, for the first time, results of the anti-cholinesterase properties of white winemaking byproducts and reports the potential suitability of cyclic voltammetry to evaluate the phenolic extraction process from these byproducts with different solvents.

## Figures and Tables

**Table 1:** Phenolic compounds identified and quantified (mg/100 g) in seeds, skins, stems and pomace extracts obtained with different solvents (ethanol, ethanol/water and water).

	Seeds			Skins			Stems			Pomace		
	Ethanol	Ethanol/ Water	Water	Ethanol	Ethanol/ Water	Water	Ethanol	Ethanol/ Water	Water	Ethanol	Ethanol/ Water	Water
**Phenolic acids**												
Gallic acid	7.42 ± 0.39 a	16.66 ± 0.30 b	41.45 ± 0.27 c	3.88 ± 0.27 a	4.18 ± 0.40 a	1.65 ± 0.28 b	3.70 ± 0.02 a	12.36 ± 1.47 b	14.98 ± 5.10 b	6.57 ± 0.08 a	17.85 ± 1.10 b	12.65 ± 0.23 c
Protocatechic acid	2.61 ± 0.00 a	3.66 ± 0.07 b	1.54 ± 0.04 c	2.65 ± 0.33 a	2.62 ± 0.30 a	5.22 ± 0.91 b	2.15 ± 0.06 a	5.22 ± 0.67 b	2.64 ± 0.26 a	2.88 ± 0.11 a	5.72 ± 0.42 b	2.82 ± 0.00 a
**Flavanols**												
Catechin	22.06 ± 2.04 a	33.04 ± 6.25 b	26.73 ± 2.33 a,b	3.31 ± 4.67 a	6.81 ± 0.29 a	NQ	NQ	23.42 ± 1.01 a	6.90 ± 0.19 b	7.98 ± 0.07 a	17.45 ± 0.22 b	4.72 ± 0.40 c
Epicatechin	27.47 ± 0.17 a	40.02 ± 2.04 b	23.75 ± 2.07 c	NQ	6.22 ± 0.97	NQ	NQ	17.09 ± 0.13 a	6.76 ± 0.00 b	6.59 ± 0.00 a	16.75 ± 0.77 b	5.91 ± 0.76 a
Pc B1	22.53 ± 3.24 a,b	29.36 ± 6.32 a	18.09 ± 1.84 b	2.96 ± 0.00 a	9.53 ± 0.06 b	NQ	NQ	28.33 ± 2.90 a	10.38 ± 5.79 b	8.44 ± 0.14 a	18.07 ± 0.44 b	7.42 ± 0.40 c
**Flavonols**												
Q-3-*O*-rutin	0.54 ± 0.18 a	0.95 ± 0.03 b	0.30 ± 0.06 c	5.11 ± 0.21 a	6.28 ± 1.39 a	0.23 ± 0.03 b	0.30 ± 0.01 a	6.62 ± 0.67 b	0.59 ± 0.13 a	1.54 ± 0.12 a	5.77 ± 1.05 b	0.35 ± 0.06 c
Q-3-*O*-gluc	1.19 ± 0.36 a	3.76 ± 0.07 b	1.18 ± 0.18 a	28.97 ± 5.46 a	48.85 ± 6.54 b	0.53 ± 0.14 c	0.77 ± 0.01 a	45.05 ± 7.73 b	5.64 ± 1.97 a	10.87 ± 0.50 a	47.81 ± 5.38 b	2.91 ± 0.01 c
K-3-*O*-gluc	0.20 ± 0.05 a	0.49 ± 0.01 b	0.18 ± 0.00 a	6.90 ± 1.10 a	7.38 ± 0.03 a	4.77 ± 0.00 b	NQ	3.73 ± 0.47 a	0.56 ± 0.03 b	1.87 ± 0.03 a	6.18 ± 0.46 b	0.43 ± 0.00 c

Each value represents the mean (*n* = 3) ± SD. For the same byproduct, values in the same row followed by different letters indicate significant differences between extracts obtained with different solvents by Tukey’s test (*p* < 0.05). NQ: not quantified. Pc, proanthocyanidin; Q, quercetin; K, kaempferol; rutin, rutinoside; gluc, glucoside; glucu, glucuronide.

**Table 2:** Antioxidant activity (mmol TE/100 g) measured by 2,2-diphenyl-1-picrylhydrazyl (DPPH) and Oxygen Radical Absorbance Capacity (ORAC) assays in seeds, skins, stems and pomace extracts obtained with different solvents (ethanol, ethanol/water and water).

	DPPH	ORAC
**Seeds**		
Ethanol	38.01 ± 2.59 a	20.44 ± 2.50 a
Ethanol/water	50.44 ± 4.32 b	45.87 ± 8.12 b
Water	7.96 ± 5.30 c	25.39 ± 0.20 a
**Skins**		
Ethanol	0.77 ± 0.03 a	24.38 ± 1.24 a
Ethanol/water	34.96 ± 2.47 b	103.50 ± 15.36 b
Water	3.07 ± 0.08 a	68.01 ± 12.93 c
**Stems**		
Ethanol	1.65 ± 0.10 a	5.59 ± 1.04 a
Ethanol/water	28.10 ± 1.49 b	33.85 ± 2.25 b
Water	4.02 ± 0.10 c	12.26 ± 0.30 c
**Pomace**		
Ethanol	5.20 ± 0.27 a	21.92 ± 3.16 a
Ethanol/water	35.43 ± 3.24 b	83.53 ± 4.89 b
Water	3.08 ± 0.04 a	37.85 ± 3.21 c

Each value represents the mean (*n* = 3) ± SD. For the same byproduct, values in the same column followed by different letters indicate significant differences between extracts obtained with different solvents by Tukey’s test (*p* < 0.05).

**Table 3:** Electrochemical parameters extracted from the cyclic voltammetry curves of the seeds, skins, stems and pomace extracts obtained with different solvents (ethanol, ethanol/water and water).

		Peak a		Peak c	
	Q	E_p,a_	I_p,a_	E_p,c_	I_p,c_
**Seeds**					
Ethanol	2.03 ± 0.19 a	0.46 ± 0.00 a	2.66 ± 0.39 a	0.32 ± 0.00 a	−1.16 ± 0.20 a
Ethanol/water	2.15 ± 0.00 a	0.48 ± 0.00 b	2.79 ± 0.08 a	0.31 ± 0.00 a	−1.13 ± 0.02 a
Water	2.03 ± 0.07 a	0.41 ± 0.00 c	3.00 ± 0.14 b	0.35 ± 0.00 b	−1.49 ± 0.03 b
**Skins**					
Ethanol	1.06 ± 0.04 a	0.48 ± 0.00 a	1.31 ± 0.04 a	0.45 ± 0.00 a	−0.43 ± 0.05 a
Ethanol/water	1.73 ± 0.03 b	0.49 ± 0.00 b	2.25 ± 0.15 b	0.27 ± 0.00 b	−1.06 ± 0.15 b
Water	1.11 ± 0.08 a	0.44 ± 0.00 c	1.14 ± 0.09 a	0.34 ± 0.00 c	−0.81 ± 0.10 c
**Stems**					
Ethanol	1.65 ± 0.01 a	0.41 ± 0.01 a	1.63 ± 0.05 a	0.27 ± 0.00 a	−0.79 ± 0.01 a
Ethanol/water	2.22 ± 0.02 b	0.47 ± 0.00 b	2.45 ± 0.01 b	0.29 ± 0.00 a	−1.17 ± 0.01 b
Water	1.46 ± 0.01 c	0.42 ± 0.00 a	1.56 ± 0.03 a	0.29 ± 0.00 a	−0.86 ± 0.00 c
**Pomace**					
Ethanol	1.34 ± 0.02 a	0.49 ± 0.00 a	2.21 ± 0.01 a	0.27 ± 0.00 a	−0.95 ± 0.02 a
Ethanol/water	1.48 ± 0.02 b	0.48 ± 0.00 b	2.02 ± 0.00 b	0.27 ± 0.00 a	−0.98 ± 0.03 a
Water	0.72 ± 0.00 c	0.42 ± 0.01 a	1.23 ± 0.03 c	0.33 ± 0.00 b	−0.67 ± 0.00 c

Each value represents the mean (*n* = 3) ± SD. For the same byproduct, different letters in the same column indicate significant differences by Tukey’s test (*p* < 0.05). E_p,a_ (the anodic peak potential) and E_p,c_ (the cathodic peak potential) are expressed as V, and I_p,a_ (the anodic peak current) and I_p,c_ (the cathodic peak current) as μA. Q: the anodic current area.
